# Characterization and distribution of an oncofetal antigen (M2A antigen) expressed on testicular germ cell tumours

**DOI:** 10.1038/sj.bjc.6690393

**Published:** 1999-05-01

**Authors:** A Marks, D R Sutherland, D Bailey, J Iglesias, J Law, M Lei, H Yeger, D Banerjee, R Baumal

**Affiliations:** Banting and Best Department of Medical Research; Departments of Medicineand Pathology, University of Toronto, 112 College Street, Toronto, Ontario, Canada M5G 1L6,

**Keywords:** germ cell neoplasia, monoclonal antibodies, oncofetal antigen

## Abstract

M2A antigen is an oncofetal antigen associated with germ cell neoplasia, present in testis on fetal gonocytes and re-expressed on carcinoma in situ (CIS) and germ cell tumours. We developed a panel of monoclonal antibodies (mAb), M2A (IgG2a), D1-26 (IgG2b) and D2-40 (IgG1), to this antigen in order to characterize its structure and study its distribution among germ cell tumours. M2A antigen was purified by sequential lectin and antibody affinity chromatography and characterized as a monomeric *M*_r_ 40 000 surface sialoglycoprotein, extensively glycosylated with O-linked carbohydrate structures, but devoid of N-linked sugars. Terminal sialic acid residues were required for reactivity with mAb M2A and D1-26, but not D2-40. Sections of 69 testicular germ cell tumours, fixed in formalin and embedded in paraffin, were stained with mAb D2-40 to examine the distribution of M2A antigen. Uniform membrane staining was observed in seminomas, and focal staining in 69% of embryonal carcinomas, 29% of teratomas and 25% of yolk sac tumours. CIS in the vicinity of all germ cell tumours also displayed uniform membrane staining. The characterization of M2A antigen, and the development of mAb which react with it in conventionally preserved archival specimens, provide important initiatives to study the origin and progression of germ cell neoplasia. © 1999 Cancer Research Campaign


*British Journal of Cancer* (1999) **80**(3/4), 569–578
© 1999 Cancer Research Campaign
Article no. bjoc.1998.0393

### Characterization and distribution of an oncofetal antigen (M2A antigen) expressed on testicular germ cell tumours

**A Marks[1], DR Sutherland[2], D Bailey[3], J Iglesias[1], J Law[1], M Lei[1], H Yeger[3], D Banerjee[3] and R Baumal[3]**

[1]Banting and Best Department of Medical Research and Departments of [2]Medicine and [3]Pathology, University of Toronto, 112 College Street, Toronto, Ontario, Canada M5G 1L6

**Summary** M2A antigen is an oncofetal antigen associated with germ cell neoplasia, present in testis on fetal gonocytes and re-expressed on carcinoma in situ (CIS) and germ cell tumours. We developed a panel of monoclonal antibodies (mAb), M2A (IgG2a), D1-26 (IgG2b) and D2-40 (IgG1), to this antigen in order to characterize its structure and study its distribution among germ cell tumours. M2A antigen was purified by sequential lectin and antibody affinity chromatography and characterized as a monomeric $M_r$ 40 000 surface sialoglycoprotein, extensively glycosylated with O-linked carbohydrate structures, but devoid of N-linked sugars. Terminal sialic acid residues were required for reactivity with mAb M2A and D1-26, but not D2-40. Sections of 69 testicular germ cell tumours, fixed in formalin and embedded in paraffin, were stained with mAb D2-40 to examine the distribution of M2A antigen. Uniform membrane staining was observed in seminomas, and focal staining in 69% of embryonal carcinomas, 29% of teratomas and 25% of yolk sac tumours. CIS in the vicinity of all germ cell tumours also displayed uniform membrane staining. The characterization of M2A antigen, and the development of mAb which react with it in conventionally preserved archival specimens, provide important initiatives to study the origin and progression of germ cell neoplasia.

**Keywords:germ cell neoplasia; monoclonal antibodies; oncofetal antigen:**

Testicular germ cell tumours (TGCT) provide a unique model for correlating tumour progression with biochemical changes. While primarily gonadal, germ cell tumours also arise occasionally from germ cell nests remaining in the mediastinum, retroperitoneum and midline of the brain (Goss et al, 1994). Histologically, these tumours can be divided into two broad classes, seminoma (SE), which comprise approximately half of these tumours, and other histological subtypes, including embryonal carcinoma (EC), immature and mature teratoma (IT and MT), yolk sac tumour (YS), choriocarcinoma (CC) and mixed germ cell tumours (MGCT). All of the above invasive and potentially metastatic malignancies are preceded by a form of non-invasive intratubular field disease, testicular carcinoma in situ (CIS), which invariably progresses over a period of up to 10 years to an invasive malignancy (Skakkebæk and Bertheleson, 1981; Von der Maase et al, 1986, 1987; Skakkebæk et al, 1987). Although the incidence of TGCT peaks between the second and fourth decade (Nicholson and Harland, 1995), the presence of shared surface markers among primordial fetal gonocytes, CIS and TGCT, but absent in normal adult testis, supports the speculation that the initial transforming event leading to germ cell neoplasia occurs prenatally in a fetal gonocyte (Skakkebæk et al, 1987; Jørgensen et al, 1995). Specifically, fetal gonocytes, CIS and TGCT express placental alkaline phosphatase, the c-*kit* proto-oncogene protein product (a receptor for stem cell factor), and two independent mucin molecules defined by their reactivity with mouse monoclonal antibodies (mAb) M2A and TRA-1-60 respectively (Jacobson and Norgaard-Pedersen, 1984; Bailey et al, 1986, 1991; Giwercman et al, 1988, 1993; Strohmeyer et al, 1991; Rajpert-De Meyts and Skakkebæk, 1994; Jørgensen et al, 1995; Rajpert-De Meyts et al, 1996). While the occasional observation of CIS during early childhood is consistent with the notion that germ cell neoplasia originates from a prenatal event (Skakkebæk et al, 1987), the possibility that a genetic programme of oncofetal antigen re-expression is induced upon post-natal transformation, giving rise to CIS, remains open. The definition of biochemical markers associated with germ cell neoplasia, and the availability of reagents to examine archival collections of gonadal and extra-gonadal tissues for the expression of these markers, would be important for addressing the issues of latency between malignant transformation, development of CIS and progression of CIS to an invasive and potentially metastatic malignancy.

We have previously described mAb M2A which reacted with a novel oncofetal membrane antigen present on fetal gonocytes, CIS and primary testicular and extratesticular SE (Bailey et al, 1986, 1988, 1991; Giwercman et al, 1988; Jørgensen et al, 1995; Meng et al, 1996). This antigen was absent in normal adult testis. M2A antigen was found as well on dysgerminoma (DG) of the ovary, the female counterpart of SE (Bailey et al, 1986). In addition to its importance as a marker of progression of testicular neoplasia, M2A antigen was also shown to be a developmental marker of human Sertoli cells, present on immature Sertoli cells until puberty, and lost during their transition to a mature adult phenotype (Baumal et al, 1989).

In order to characterize this antigen, we have produced two additional mAb, D1-26 (Ig2b) and D2-40 (IgG1), which have a similar specificity to M2A, and, in addition, react with formalin

*Received 14 April 1998*
*Revised 30 September 1998*
*Accepted 28 September 1998*

*Correspondence to:* A Marks and B5-fixed tissues. Using this panel of mAb, we show that M2A antigen is an O-linked sialoglycoprotein containing a simple mucin-type carbohydrate epitope. Furthermore, since mAb M2A does not react with specimens routinely fixed in formalin during conventional preservation (Giwercman et al, 1991), we used mAb D2-40 to stain sections of 69 archival blocks of TGCT, fixed in formalin and embedded in paraffin, in order to extend our previous results on the distribution of M2A antigen. We confirm uniform membrane staining for M2A antigen in SE and CIS in the vicinity of all TGCT. We also report focal staining for M2A antigen in EC, IT and MT and YS, not previously detected in frozen sections using mAb M2A (Bailey et al, 1991).

### MATERIALS AND METHODS

### Human tumour cell lines

The following human tumour cell lines were obtained from the American Type Culture Collection (ATCC, Rockville, MD, USA): ovary – Caov-3 (ATCC HTB-75) and SK-OV-3 (ATCC HTB-77); bone – U-2 OS (ATCC HTB-96); bone marrow – KG-1a (ATCC CCL-246.1); cervix – HeLa (ATCC CCL-2). The other cell lines were generously provided by the following investigators: ovary – HEY and HOC-7 (R Buick, University of Toronto), and NIH:OVCAR-3 (RF Ozols, Fox Chase Cancer Center); bladder – RT4, MGH-U3 and T-24 (Y Fradet, Laval University); breast – MCF7 (R Buick), MDA-MB-468 (R Reilly, University of Toronto); prostate – DU 145, PC-3 and LNCaP (T Brown, University of Toronto); colon – Caco-2 (B Schimmer, University of Toronto); pharynx – KB-3-1 (M Gottesman, National Institutes of Health); melanoma – CaCL 74-36 and CaCL 78-1 (S-K Liao, McMaster University), and WM 164 (J. Hainfeld, Brookhaven National Laboratory). The cell lines were grown in monolayer culture, except for KG-1a which was grown in suspension culture, in minimal essential medium (MEM)-α (University of Toronto Media Center, Toronto, ON, Canada) supplemented with 10% heat-inactivated fetal bovine serum and L-glutamine (Gibco-BRL, Gaithersburg, MD, USA) at 37°C, in a humidified 5% carbon dioxide incubator.

### Monoclonal antibodies mAb M2A was produced by immunizing BALB/c mice with the human ovarian epithelial carcinoma cell line HEY, as previously described (Bailey et al, 1986), and D1-26 and D2-40 by immunization with DG tissue resected from a female patient. Hybridoma colonies were initially screened by enzyme-linked immunosorbent assay (ELISA) for production of mouse IgG. Hybridomas which produced IgG were screened directly for immunoperoxidase staining of sections of formalin-fixed, paraffin-embedded human SE and tonsil tissue. Hybridomas which produced mAb reacting with SE, but not tonsil, were cloned twice by limiting dilution. mAb were purified from ascitic fluid as previously described (Sheldon et al, 1986), according to protocols approved by the University of Toronto Animal Care Committee.

### Determination of binding affinities of mAb to HEY cells mAb D1-26 and D2-40 were labelled with $^{125}$I using *N*-bromo-succinimide, as described previously (Buckman et al, 1992). mAb M2A was labelled with $^{125}$I using the Bolton Hunter reagent, *N*-succinimidyl-3-(4-hydroxyphenyl)-propionate (Amersham, Oakville, ON, Canada) (Sheldon et al, 1986). The binding affinities of the mAb to HEY cells were determined by two methods: first, direct binding using a Scatchard plot, and second, displacement of binding with increasing amounts of unlabelled mAb, as previously described (Sheldon et al, 1986).

### Western blotting

The cells were harvested by trypsinization and recovered by centrifugation. The cell pellet was suspended at a concentration of 1–2 × $10^7$ cells per ml in lysis buffer, 150 mM sodium chloride (NaCl), 2 mM EDTA, 10 mM Tris-HCl, pH 8.0, containing 0.5% Nonidet P-40 and 2 mM phenylmethylsulphonyl fluoride (PMSF; Sigma, St Louis, MO, USA). After 20–30 min at 4°C, nuclei and cellular debris were removed by centrifugation at 100 000 ***g*** for 30 min. The clarified supernatants were stored at –70°C until use. Cell lysates of the following human cell lines, testicular terato-carcinoma 577, Tera-1 and Tera-2, embryonal carcinoma 833 and 1618, ovarian teratocarcinoma PA-1, and glioblastoma T98G (King et al, 1997), were kindly provided by Dr David Hogg (University of Toronto). Aliquots containing 10 μg of protein were analysed by sodium dodecyl sulphate-polyacrylamide gel electrophoresis (SDS-PAGE) in 10% slab gels under reducing conditions. Western blotting was performed as previously described (Bailey et al, 1986) except that the secondary antibody was the peroxidase-conjugated F(ab´)$_2$ fragment of goat anti-mouse IgG antibody (Jackson Immuno Research, West Grove, PA, USA) used at a 1:10 000 dilution. The blots were developed using enhanced chemiluminescence (ECL, Amersham) and exposed to X-ray film, or alternatively, by addition of the peroxidase substrate, 0.05% diaminobenzidine and 0.03% hydrogen peroxide ($H_2O_2$) in phosphate-buffered saline (PBS).

### Lectin affinity chromatography

HEY cell lysates were subjected to lectin affinity chromatography on columns of concanavalin A, *Ricinus communis* agglutinin and wheat germ agglutinin (WGA), eluted with 10% α-methyl D-mannose, 10% D-galactose and 5% *N*-acetyl-D-glucosamine, respectively, as described previously (Sutherland et al, 1988). The presence of antigen reacting with mAb M2A in the bound and the unbound fractions was assessed by Western blotting.

### Antigen purification

The protocol for antigen purification was adapted from our previously published procedures (Sutherland et al, 1988), as follows. A pellet of 2 × $10^9$ HEY cells was suspended at 4°C in 25 ml of lysis buffer and the cell lysate was prepared and stored at –70°C, as described above. Following thawing, the cell lysate was applied to a WGA-Sepharose 4B column, and the bound glycoprotein fraction was eluted with 5% *N*-acetyl-D-glucosamine in lysis buffer. The eluate was adjusted to 0.5 M NaCl, and subjected to affinity chromatography on an immunoaffinity column, prepared by cross-linking 10 mg of mAb M2A to 1 ml protein A-Sepharose 4B (Pharmacia, Baie d'Urfé, Quebec, Canada), as described previously (Schneider et al, 1982). The column was washed extensively, first with lysis buffer, then with the same buffer containing 0.5% sodium deoxycholate and 0.5% SDS, and finally,

*© Cancer Research Campaign 1999*

with the same buffer containing 0.5% of n-octylglucoside (Boehringer Mannheim, Montreal, Quebec, Canada). The bound antigen was eluted with 50 mM diethylamine, pH 11.5, containing 0.5% sodium deoxycholate, neutralized, concentrated and analysed by SDS-PAGE in 8.5% slab gels under non-reducing conditions. The protein bands were visualized by silver staining using a commercial reagent (Bio-Rad, Mississauga, ON, Canada), and the proteins reacting with mAb M2A were identified by Western blotting.

### Enzymatic digestion

*Vibrio cholera* neuraminidase, *Pasteurella haemolytica* O-sialoglycoprotease and *Bacillus cereus* phosphatidylinositol phospholipase C digestions were performed on whole cells and purified antigen preparations. HEY cells were harvested by incubation in PBS containing 20 mM EDTA and resuspended at a concentration of $1 \times 10^7$ per ml in PBS. Aliquots of 100 µl were treated with 5 units of neuraminidase (BDH, Mississauga, ON, Canada), 5 µl of O-sialoglycoprotease (Cederlane, Hornby, ON, Canada), or 5 µl of phosphatidylinositol phospholipase C (Boehringer Mannheim), for 30 min at 37°C. The cells were washed, incubated with mAb M2A, D1-26, or D2-40, followed by a fluorescein isothiocyanate-conjugated $F(ab')_2$ fragment of goat anti-mouse Ig antibody, and analysed by flow cytometry using a BD FACScan flow cytometer (Becton Dickinson Immunocytometry Systems, San Jose, CA, USA).

For treatment of partially purified antigen preparations, 10 µl (0.15 µg) aliquots of the material eluted from the M2A-Sepharose column were mixed with 1 µl of 50 mM sodium acetate buffer, pH 5.4, and incubated with 0.1–2 units of neuraminidase, or 5 µl of O-sialoglycoprotease, for 15 min to 3 h at 37°C. The reactions were stopped by boiling, and the treated aliquots were analysed by Western blotting.

### Analysis of $^{125}$I-labelled surface proteins

HEY cell surface proteins were labelled enzymatically with $^{125}$I using lactoperoxidase, as described previously (Sutherland et al, 1988). Cell lysates were prepared as described above and immunoprecipitated with mAb M2A and protein A-Sepharose beads (Bailey et al, 1986). The $^{125}$I-labelled protein was released from the immunoprecipitate by boiling for 5 min in 100 µl of sample buffer, 0.1 M Tris, pH 6.8, containing 10% glycerol and 2% SDS (non-reduced protein), or of sample buffer containing 2% 2-mercaptoethanol (reduced protein), analysed by SDS-PAGE in 10% polyacrylamide slab gels, and visualized by autoradiography, as described previously (Sutherland et al, 1984).

Peanut agglutinin (PNA) chromatography of desialylated immunoprecipitated protein was performed as described previously (Sutherland et al, 1988). The bound protein fraction was analysed by SDS-PAGE and autoradiography.

For endoglycosidase digestion, the M2A antigen was eluted from the gel, recovered by precipitation in 10% trichloroacetic acid, divided into aliquots and digested with endoglycosidase H (Eli Lilly, Indianapolis, IN, USA), peptide:*N*-glycosidase F (Boehringer Mannheim), or sham-digested, as described previously (Sutherland et al, 1988). The treated protein was recovered by precipitation in 10% trichloroacetic acid, and analysed by SDS-PAGE and autoradiography.

### Immunoperoxidase staining

Sections of archival collections of TGCT were obtained through the Departments of Pathology of The Toronto Hospital, General Division, and The Princess Margaret Hospital (Toronto, ON, Canada). Immunoperoxidase staining of tissue sections was performed as previously described (Bailey et al, 1986), with the following modifications. Following incubation with hybridoma supernatant or purified mAb (0.1–10 µg $ml^{-1}$), the sections were incubated with biotinylated goat anti-mouse IgG antibody (Dako, Carpinteria, CA, USA) at a 1:400 dilution followed by a horseradish peroxidase–avidin conjugate (Zymed, San Francisco, CA, USA) at a 1:600 dilution. For colour development, the sections were incubated with 0.05% diaminobenzidine and 0.03% $H_2O_2$ in PBS.

### RESULTS

### Production of mAb

Previously derived mAb M2A (IgG2a) was found to react with an oncofetal testicular antigen in frozen sections and tissues preserved in specialized fixatives, such as Stieve's, Bouin's and Cleland's reagents (Bailey et al, 1986; Giwercman et al, 1991). In order to generate additional mAb for the biochemical characterization of M2A antigen and its immunohistochemical detection in archival specimens, we immunized mice with DG and screened hybridoma supernatants for reactivity with sections of formalin-fixed, paraffin-embedded SE. The underlying rationale for this approach was twofold. First, it was expected to yield mAb directed to antigens (including M2A antigen) shared by SE and DG, and second, the direct screen with SE sections, as specified above, would ensure that the selected mAb reacted with conventionally preserved archival collections of tissues. In fact, two mAb, D1-26 (IgG2b) and D2–40 (IgG1), were successfully obtained using this approach. Of the two, D2-40 reacted strongly with sections of formalin-fixed, paraffin-embedded tissues, whereas D1-26 reacted only weakly and inconsistently. Therefore, D2-40 was used for all subsequent immunohistochemical staining of conventionally preserved archival material. Both D1-26 and D2-40 reacted strongly with tissues preserved in B5 fixative, a mercuric-type reagent used to preserve the immunoreactivity of membrane antigens. In contrast, mAb M2A did not react with tissues fixed in formalin and only weakly with those fixed in B5.

### Distribution of M2A antigen in fetal and neoplastic testicular tissue mAb D1-26 and D2-40 stained testicular SE fixed in B5 or formalin, respectively, with strong accentuation of tumour cell membranes (Figure 1 B,C), similar to that observed with M2A in frozen sections (Figure 1A). Paranuclear staining was also seen with mAb D2-40 (Figure 1D), indicative of the location of the antigen in the paranuclear Golgi network where glycoproteins are processed prior to their insertion into the membrane. D2-40 also stained sections of DG used for the original immunization of mice (Figure 1E), EC (Figure 1F), CIS (Figure 1G) and fetal testis (Figure 1H). There was no staining of uninvolved adult testis in the vicinity of germ cell neoplasia (Figure 1G). There was uniform membrane staining of tumour cells in SE, DG and CIS (Figure 1 C,E,G), whereas staining in EC (Figure 1F) was focal in

© Cancer Research Campaign 1999

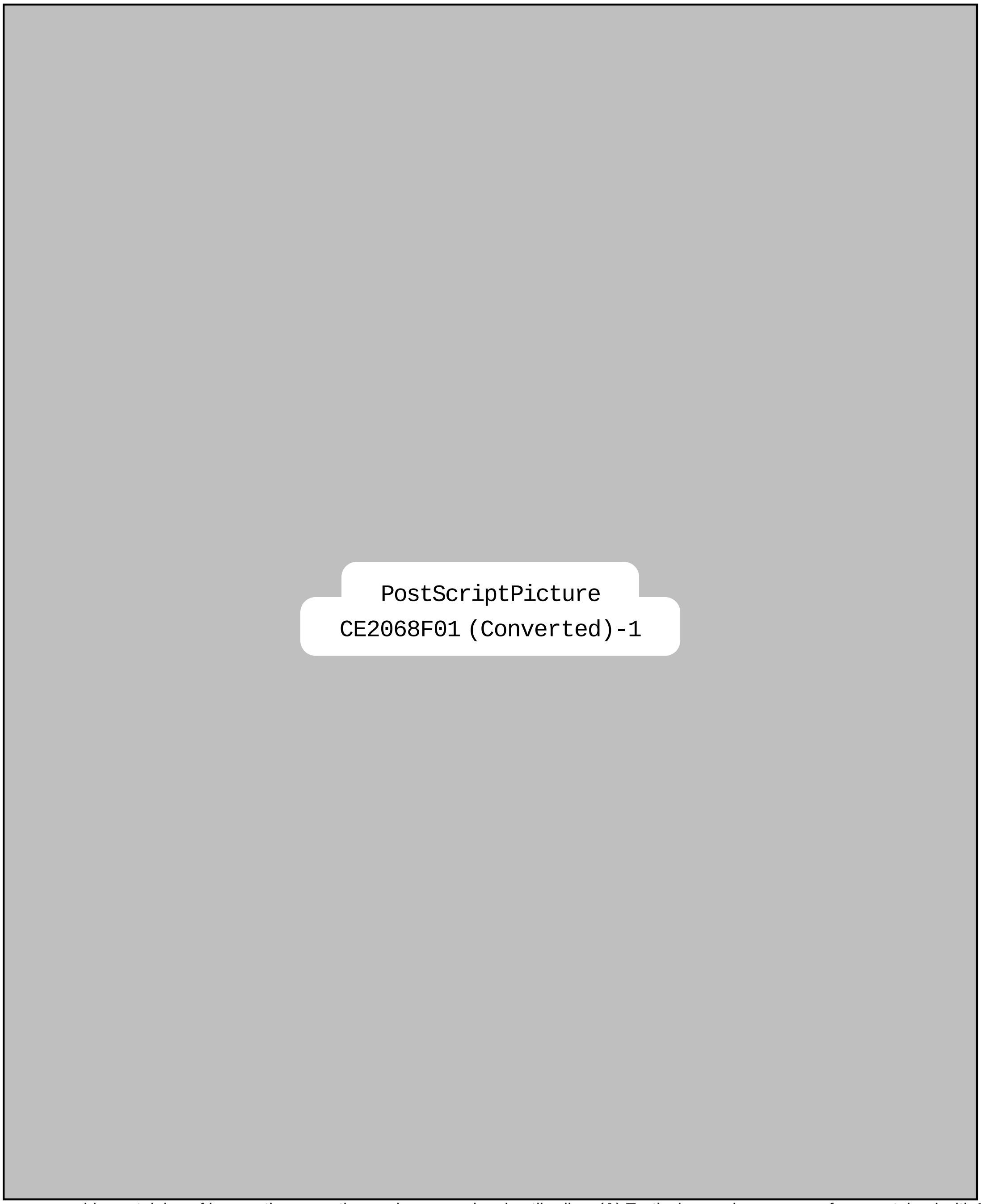

**Figure 1** Immunoperoxidase staining of human tissue sections using monoclonal antibodies. (**A**) Testicular seminoma, snap-frozen, stained with M2A, 10 $\mu$g ml$^{-1}$, $\times$ 190. (**B**) Testicular seminoma fixed in B5, stained with D1–26, 0.1 $\mu$g ml$^{-1}$, $\times$ 190. (**C–H**) Tissues fixed in formalin, stained with D2-40, 0.1 $\mu$g ml$^{-1}$. (**C**) Testicular seminoma $\times$ 100. (**D**) Testicular seminoma showing paranuclear staining (arrows), $\times$ 150. (**E**) Ovarian dysgerminoma, $\times$ 190. (**F**) Embryonal carcinoma, $\times$ 150. Mitotic figures are indicated by arrows. (**G**) Adult testis with CIS, showing positive staining of CIS (left) and absence of staining of uninvolved tubule (right), $\times$ 120. (**H**) Fetal testis, $\times$ 190

distribution, concentrated at the lumenal surface of the membrane in the tumour cells which lined glands. D2-40 also stained primary extratesticular SE, including pineal (2/2) and mediastinal (2/2). This pattern of reactivity with testicular and extratesticular SE, DG, CIS and fetal, but not adult, testis is the same as that previously observed for mAb M2A in these tissues. In earlier studies on a limited number of cryopreserved TGCT, mAb M2A stained only CIS and SE, but not the other types of TGCT (Bailey et al, 1991). Since mAb D2-40 reacted with formalin-fixed, paraffin-embedded material, it was possible to extend these studies by examining an archival collection of 69 TGCT for the distribution of M2A antigen. This collection included 41 pure TGCT (35 SE, 5 EC and 1 IT), and 28 mixed germ cell tumours (MGCT). The results of this survey are summarized in Table 1.

When the results obtained with pure TGCT and the corresponding components of MGCT were combined, mAb D2-40 was found to stain 98% of SE, 69% of EC, 29% of IT and MT, and 25% of YS. There was no difference in staining between IT and MT.

 *© Cancer Research Campaign 1999*

**Table 1** Reactivity of mAb D2-40 with testicular germ cell tumours

| Specimen type | Positive staining[a] |
|---|---|
| Pure germ cell tumour | |
| SE | 34/35 |
| EC | 1/5 |
| IT and MT | 0/1 |
| MGCT component | |
| SE | 12/12 |
| EC | 17/21 |
| IT and MT | 5/16 |
| YS | 1/4 |
| CC | 0/2 |
| CIS associated with: | |
| SE | 21/21 |
| EC | 1/1 |
| MGCT | 13/13 |

[a]Ratios indicate number of specimens positively stained over the total number examined.

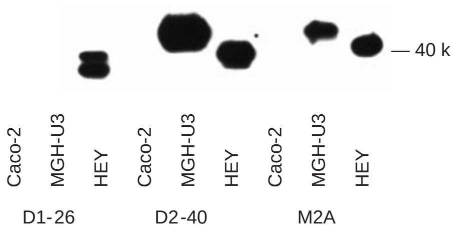

**Figure 2** Western blot analysis of human cancer cell line lysates. Cell lysates were prepared and analysed by Western blotting, using enhanced chemiluminescence. The cell lines used to prepare the lysates are indicated below the individual lanes: Caco-2 – colon, MGH-U3 – bladder, HEY – ovary. The antibodies used for Western blotting are specified below each Western blot. The position of the $M_r$ 40k antigen is indicated

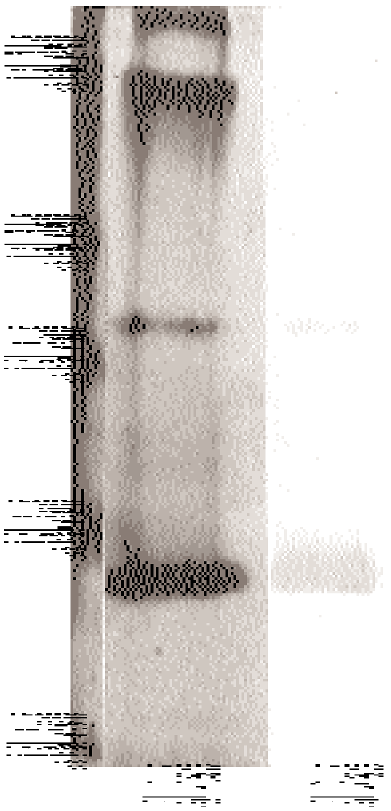

**Figure 3** Electrophoretic analysis of purified antigen. The antigen recognized by mAb M2A was purified from HEY cell lysate by sequential affinity chromatography on WGA-Sepharose and M2A-Sepharose. The fraction eluted from M2A-Sepharose was analysed by SDS-PAGE under non-reducing conditions. (**A**) The proteins bands on the gel were visualized by silver staining. (**B**) The protein bands reacting with mAb M2A were demonstrated by Western blotting, using the peroxidase substrate diaminobenzidine. The size of the molecular weight markers ($M_r \times 10^{-3}$) is indicated

The only SE specimen that did not stain was an anaplastic SE, a tumour type that has been previously noted to display an aberrant pattern of antigen expression (Rajpert-De Meyts et al, 1996). There was 100% staining of CIS in association with both seminomatous and non-seminomatous TGCT. Whereas uniform membrane staining was observed in SE and CIS, the staining in EC, IT, MT and YS was focal in distribution, concentrated at the lumenal surface of the membrane in the tumour cells which lined glands. There was no staining of the uninvolved testicular tubules in the vicinity of tumour cells in any of the specimens.

### Reactivity of mAb with human tumour cell lines

The reactivity of the mAb with human tumour cell lines was determined by Western blotting on cell lysates. A representative Western blot is shown in Figure 2. In ovarian cancer HEY cell lysates, M2A and D2-40 reacted with an $M_r$ 40 000 antigen, and D1-26 reacted with a doublet form of the antigen. In bladder cancer MGH-U3 cells, M2A and D2-40, but not D1-26, reacted with a slightly higher molecular weight form of the antigen. Neither M2A, D2-40 nor D1-26 reacted with colon cancer Caco-2 cells. The reactivity of mAb M2A, D2-40 and D1-26 with a panel of human cancer cell lines, determined by Western blotting, is summarized in Table 2. All three mAb reacted with three ovarian epithelial carcinoma cell lines, HEY, Caov-3 and SK-OV-3, and the osteosarcoma cell line U-2 OS. Among germ cell tumour cell lines, D2-40, but not M2A or D1-26, reacted with the ovarian teratocarcinoma PA-1, the testicular teratocarcinomas Tera-1 and Tera-2, and the embryonal carcinoma 833. There was no reactivity with the testicular teratocarcinoma 577 and the embryonal carcinoma 1618. In addition, M2A and D2-40, but not D1-26, reacted with the bladder transitional cell carcinoma cell lines MGH-U3 and RT-4. There was no reactivity with any of the other cell lines derived from a wide spectrum of human cancers, including ovary, bladder, breast, prostate, melanoma, cervix, colon, pharynx, brain and bone marrow.

The binding affinities of M2A, D1-26 and D2-40 to HEY cells were found to be $2 \times 10^9$ M$^{-1}$, $2 \times 10^8$ M$^{-1}$, and $2 \times 10^8$ M$^{-1}$, respectively, on the basis of Scratchard plot and binding displacement analyses.

### Antigen purification

Preliminary lectin affinity chromatography experiments indicated that the antigen recognized by the mAb bound to WGA, but not to concanavalin A or *R. communis* agglutinin. The antigen was purified from HEY cell lysates by sequential affinity chromatography

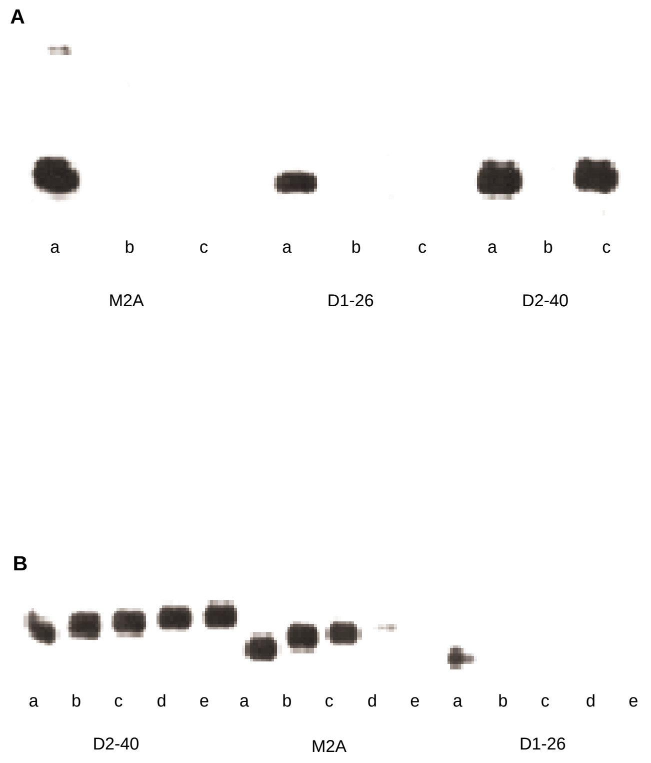

**Figure 4** (**A**) Western blot analysis of purified antigen following enzymatic digestion. The purified antigen preparation was analysed for reactivity with mAb M2A, D1–26 and D2–40 by Western blotting, using enhanced chemiluminescence, either untreated, or following digestion with O-sialoglycoprotease (5 μl), or neuraminidase (2 units), for 3 h at 37°C. The mAb used is indicated below each Western blot. Individual lanes were treated as follows: (a) no treatment, (b) O-sialoglycoprotease, (c) neuraminidase. (**B**) Western blot analysis of purified antigen following digestion with increasing amounts of neuraminidase. The purified antigen preparation was digested with increasing amounts of neuraminidase, and analysed by Western blotting, using enhanced chemiluminescence. The mAb used is indicated below each Western blot. The amounts of neuraminidase used and the lengths of digestion in individual lanes were as follows: (a) 0; (b) 0.1 unit, 15 min; (c) 0.1 unit,1 h; (d) 0.25 unit, 1 h; (e) 2 units, 1 h

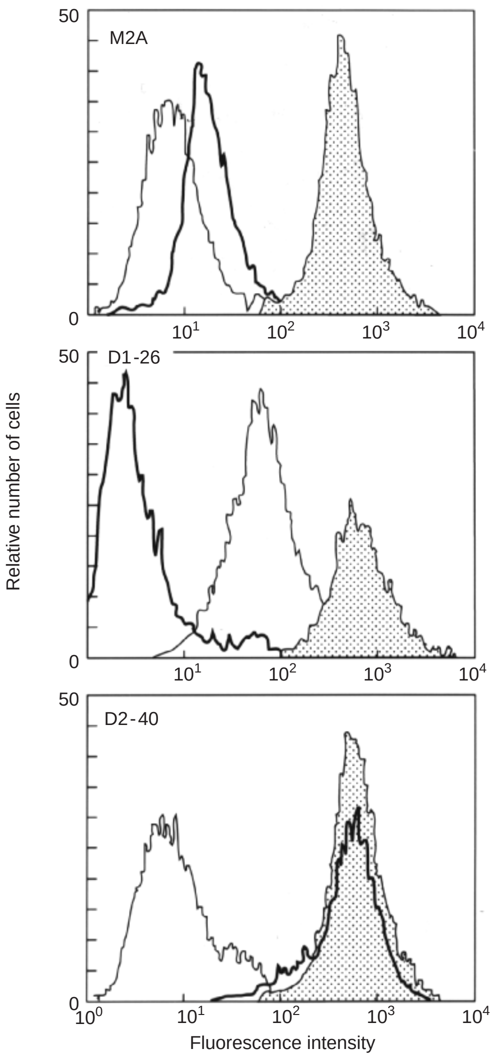

**Figure 5** Flow cytometric analysis of HEY cells following enzymatic digestion. HEY cells were digested with neuraminidase or O-sialoglycoprotease, then stained by indirect immunofluorescence with mAb M2A, D1–26, or D2–40 and analysed by flow cytometry, as indicated in the individual panels. The shaded peaks represent control cells not subjected to enzymatic digestion. Cells digested with O-sialoglycoprotease prior to staining with the mAb are indicated by a thin solid line, and those digested with neuraminidase by a thick solid line on WGA-Sepharose and M2A-Sepharose. The purified antigen preparation contained four protein bands resolved on SDS-PAGE and visualized by silver staining (Figure 3, lane A). The major band at $M_r$ 40 000 corresponded to a band stained by M2A on a Western blot (Figure 3, lane B), representing the purified antigen monomer. A higher molecular weight band at $M_r$ 80 000 was also visualized on a Western blot following staining with M2A, but not when the secondary peroxidase-conjugated anti-mouse IgG reagent alone was used. This band likely represents an antigen dimer. Two additional contaminating high molecular weight bands ($M_r$ > 200 000) were also seen on the silver-stained gel, but not on the Western blot (Figure 3).

### Enzymatic digestion of purified antigen and whole cells mAb M2A, D1-26 and D2-40 reacted with the purified $M_r$ 40 000 antigen on Western blotting (Figure 4A, lane a). The reactivity of the three mAb with the antigen was destroyed by prior treatment with O-sialoglycoprotease (Figure 4A, lane b), indicating that the antigen was an O-linked glycoprotein. The reactivity with M2A and D1-26, but not D2-40, was also destroyed by treatment with neuraminidase (Figure 4A, lane c), showing that a terminal sialic acid residue in the epitope was required for reactivity with M2A and D1-26.

Sequential digestion of the purified antigen preparation with increasing amounts of neuraminidase demonstrated a slower migration of the antigen with progressive removal of negatively charged sialic acid residues (Figure 4B). This was seen using mAb D2-40, which reacted with the desialylated antigen, and M2A, which still reacted with the antigen after partial removal of the sialic acid residues. In contrast, the reactivity of D1-26 with the antigen was exquisitely sensitive to neuraminidase digestion, and was lost after removal of sialic acid under the mildest conditions used for digestion with the enzyme.

The above results were extended using HEY cell cultures by performing enzymatic digestions on whole cells and detection of

 *© Cancer Research Campaign 1999*

the surface antigen by flow cytometry (Figure 5). Reactivity with all three mAb was sensitive to digestion with O-sialoglycoprotease, although reactivity with D1-26 was less sensitive to digestion with the enzyme than reactivity with M2A and D2-40 (Figure 5, thin solid line). On the other hand, reactivity with D1-26 was most sensitive to digestion with neuraminidase, reactivity with M2A was partially sensitive, and reactivity with D2-40 was resistant to digestion with the enzyme (Figure 5, thick solid line), confirming the results obtained using Western blot analysis.

Reactivity of HEY cells with mAb M2A, as assessed by flow cytometry, was not affected by treatment with phosphatidylinositol phospholipase C, suggesting that the antigen was not anchored in the membrane through a glycophosphatidylinositol linkage (data not shown).

### Analysis of $^{125}$I-labelled surface proteins

HEY cell surface proteins were labelled with $^{125}$I using lactoperoxidase, immunoprecipitated indirectly using mAb M2A and protein A-Sepharose, dissociated from the immune complex under reducing and non-reducing conditions, and analysed by SDS-PAGE. A single $M_r$ 40 000 band was visualized on the autoradiogram under both reducing and non-reducing conditions (Figure 6A, lanes 1 and 2), corresponding to the monomeric form of the antigen demonstrated previously by Western blotting (Figures 2 and 3).

Two approaches were used to further characterize the carbohydrate structure of the antigen. In the first approach, the $^{125}$I-labelled protein was dissociated from the immunoprecipitate, digested with neuraminidase to remove sialic acid and incubated with PNA-Sepharose beads. The protein bound to the lectin was dissociated from the beads and analysed by SDS-PAGE. A single protein band was visualized on the autoradiogram, migrating more slowly ($M_r$ 42 000) than the antigen immunoprecipitated without treatment with neuraminidase (Figure 6A, lane 3).

In the second approach, the $^{125}$I-labelled protein was again immunoprecipitated, dissociated from the immunoprecipitate and subjected to SDS-PAGE as described above. The $^{125}$I-labelled antigen was eluted from the polyacrylamide gel, incubated with buffer alone, endoglycosidase H, or peptide:*N*-glycosidase F, and re-analysed by SDS-PAGE (Figure 6B, lanes 6–8). The antigen was not cleaved by digestion with either of these enzymes.

### DISCUSSION

mAb M2A was found to react with a membrane antigen present on fetal gonocytes, CIS and SE in testicular tissues preserved by freezing or immersion in specialized fixatives (Bailey et al, 1986, 1991; Giwercman et al, 1988, 1991; Jørgensen et al, 1995). In order to extend these results on the distribution of M2A antigen and elucidate its biochemical structure, our experimental goal was to produce mAb with a similar specificity to M2A which also reacted with formalin-fixed, paraffin-embedded material.

Since M2A antigen was present on SE and its female counterpart, DG, we used an experimental strategy to immunize mice with DG, and to screen hybridomas, derived by fusion of splenocytes of immunized mice, for production of mAb reacting with sections of formalin-fixed, paraffin-embedded SE specimens. This strategy was successful and two mAb were produced, D1-26 and D2-40, which reacted with B5- and formalin-fixed, paraffin-embedded tissues (Figure 1) with a specificity for fetal gonocytes, CIS, testicular and extratesticular SE, and DG similar to that demonstrated previously for mAb M2A (Bailey et al, 1986, 1991; Jørgensen et al, 1995). The conclusive evidence that the three mAb reacted with the same antigen was provided by purifying the $M_r$ 40 000 antigen from HEY cell lysates (Figure 3), and demonstrating that the three mAb reacted with the same purified antigen

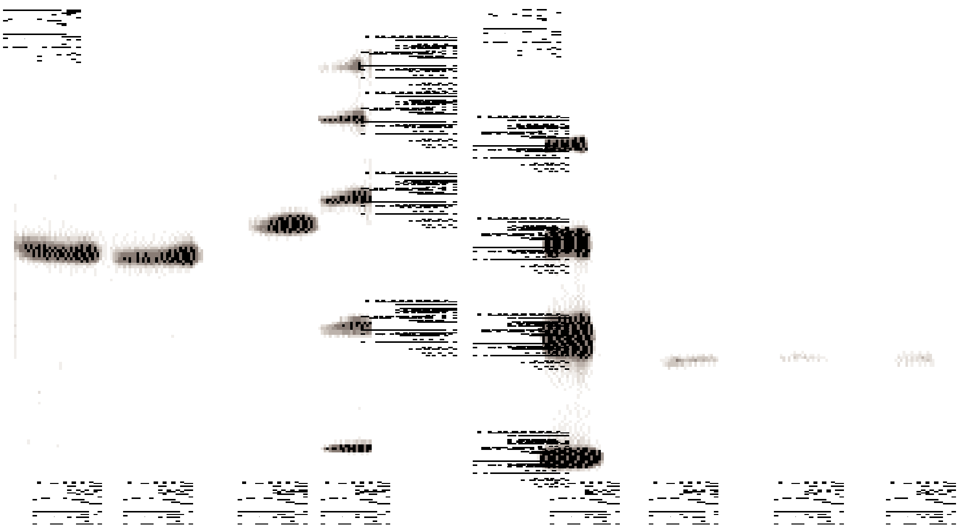

**Figure 6** Analysis of $^{125}$I-labelled surface proteins. HEY cell surface proteins were labelled with $^{125}$I, immunoprecipitated indirectly using mAb M2A and protein A-Sepharose, dissociated from the immune complex under reducing and non-reducing conditions, and analysed by SDS-PAGE in 10% polyacrylamide slab gels followed by autoradiography. (**A**) Lane 1 – non-reduced protein, lane 2 – reduced protein, lane 3 – protein dissociated from the immune complex, digested with neuraminidase, bound to PNA-Sepharose beads, and eluted from PNA-Sepharose, lane 4 – molecular weight markers with $M_r \times 10^{-3}$ indicated. (**B**) Proteins were immunoprecipitated and analysed by SDS-PAGE, as described for lane 1 above. The antigen band was eluted from the gel and divided into three aliquots which were incubated with buffer alone, endoglycosidase H, or peptide:*N*-glycosidase F, recovered by precipitation in 10% trichloroacetic acid and re-analysed by SDS-PAGE, lane 5 – molecular weight markers with $M_r \times 10^{-3}$ indicated, lane 6 – buffer alone, lane 7 – endoglycosidase H, lane 8 – peptide:*N*-glycosidase F

© Cancer Research Campaign 1999

**Table 2** Reactivity of monoclonal antibodies with human cancer cell lines[a]

| | Cancer cell line |
|---|---|
| Ovary | |
| HEY, Caov-3, SK-OV-3 | ●●●[b] |
| NIH:OVCAR-3, HOC-7 | ○○ |
| PA-1PA-1 | ◒ |
| Testis | |
| Tera-1, Tera-2, 833 | ◒◒◒ |
| 577, 1618 | ○○ |
| Bone | |
| U-2 OS | ● |
| Bladder | |
| MGH-U3, RT-4, T-24 | ◓◓○ |
| Breast | |
| MCF7, MDA-MB-468 | ○○○ |
| Prostate | |
| DU145, PC-3, LNCaP | ○○○ |
| Melanoma | |
| CaCL 78-1, WM 164 | ○○ |
| CaCL 74-36 | ○ |
| Cervix | |
| HeLa | ○ |
| Colon | |
| Caco-2 | ○ |
| Pharynx | |
| KB-3-1 | ○ |
| Brain | |
| T98G | ○ |
| Bone marrow | |
| KG-1a | ○ |

[a]Reactivity of mAb was determined on cell lysates by Western blotting.
[b]● Positive reactivity with M2A, D2-40, and D1-26; ◒ Positive reactivity with D2-40, but not M2A or D1-26; ◓ Positive reactivity with M2A and D2-40, but not M2A or D1-26; ○ No reactivity with M2A, D2-40, or D1-26.

(Figure 4). The success in selecting the hybridoma clones producing mAb D1-26 and D2-40 by screening on SE following immunization of mice with DG, a procedure completely different from that used to produce mAb M2A by immunization with HEY cells, suggests that the M2A antigen recognized by the three mAb may be an immunodominant antigen on SE and DG.

M2A antigen was expressed in several human cancer cell lines, including three ovarian epithelial carcinoma, one ovarian and two testicular teratocarcinoma, one embryonal carcinoma, one osteosarcoma and two bladder transitional carcinoma, but was absent in many other cell lines derived from a variety of human tumours (Table 2). At present, it is not known whether the expression of this antigen in a number of human cancer cell lines is indicative of a lineage-specific marker shared by the cell line and the tumour from which it was derived, or alternatively, whether the presence of the antigen represents an aberrant expression of the protein by the transformed cell line in vitro.

The presence of the antigen in the paranuclear Golgi apparatus suggested that it was a glycoprotein (Figure 1D). This suggestion was supported by variations in the molecular weight of the antigen (Figure 2) and in its recognition by the individual mAb (Table 2) in human cancer cell lines, likely reflecting differences in glycosylation. In confirmation of this suggestion, reactivity with the mAb was sensitive to digestion with O-sialoglycoprotease and neuraminidase on the basis of Western blotting and flow cytometric analyses (Figures 4 and 5), indicating that the antigen was an O-linked sialoglycoprotein exposed on the cell surface. As expected, removal of the negatively charged sialic acid residues decreased the electrophoretic mobility of the antigen (Figure 4B). Furthermore, the reactivity of the three mAb with their respective complementary carbohydrate epitopes was differentially affected by treatment with neuraminidase, D1-26 reactivity being exquisitively sensitive to the removal of sialic acid, D2-40 reactivity completely resistant, and M2A reactivity intermediate between the other two (Figures 4 and 5).

We took advantage of the surface localization of the antigen to label it with $^{125}$I using lactoperoxidase. This allowed a more direct examination of its molecular structure in its usual membrane location. Following immunoprecipitation of the $^{125}$I-labelled HEY cell surface proteins with mAb M2A and SDS-PAGE, a single protein band was visualized on the autoradiogram after dissociation of the immunoprecipitate under both reducing and non-reducing conditions (Figure 6A). This suggests that the antigen exists as a monomer and is not complexed with another membrane protein. The dimeric form, previously observed in the purified antigen preparation (Figure 3), likely is formed by aggregation during the purification procedure. Alternatively, the dimeric form in the membrane may not be accessible to iodination with lactoperoxidase.

The $^{125}$I-labelled antigen was found to be resistant to digestion with endoglycosidase H and peptide:*N*-glycosidase F, enzymes which cleave asparagine-bonded oligomannosyl cores of precursor N-linked glycoproteins and also complex-type N-linked glycans characteristic of the fully processed cell surface glycoproteins (Tarentino and Maley, 1974; Plummer et al, 1984; Sutherland et al, 1988) (Figure 6B). These results confirmed that the antigen was an O-linked glycoprotein with no N-linked sugar groups. Absence of N-linked sugar residues was also consistent with the lack of binding of the antigen to concanavalin A, which binds specifically to N-linked glycans (Wu et al, 1988), and our inability to radiolabel the antigen metabolically using the non-convertible D-[2-$^{3}$H]mannose precursor of N-linked sugars (unpublished observations).

In order to characterize further the carbohydrate structure of the antigen recognized by the mAb, we used affinity chromatography on PNA-Sepharose. PNA binds to the structure *Gal* β(1–3) *Gal*NAc-0-*Thr*/*Ser* which is found only in O-linked glycoproteins (Wu et al, 1988). When the $^{125}$I-labelled M2A immunoprecipitate was digested with neuraminidase and fractionated on PNA-Sepharose, the desialylated antigen was found to bind to the lectin. Following elution from the lectin, the desialylated antigen migrated more slowly in SDS-PAGE than the native antigen (Figure 6A), as shown previously by Western blotting (Figure 4B). Removal of sialic acid was essential for binding to PNA-Sepharose, as the native antigen did not bind to the lectin (unpublished observations). Similarly, failure of the native antigen to bind to *R. communis* agglutinin confirmed the absence of exposed galactose or *N*-acetyl galactosamine residues, and binding to WGA was also consistent with terminal sialylation (Peters et al, 1979). In addition, reactivity of the antigen with mAb M2A was destroyed by treatment with periodate, a reaction that oxidizes the terminal sialic acid residues of glycoproteins (unpublished results).

The results of enzyme digestion and lectin fractionation suggest that the antigen recognized by mAb M2A, D1-26 and D2-40 contains the simple mucin-type carbohydrate structure shown below.

 *© Cancer Research Campaign 1999*

SA α(2–3) *Gal* β(1–3) *Gal*NAc-0-*Thr*/*Ser*

| α (2–6)

SA

where SA denotes sialic acid, *Gal* galactose and *Gal*NAc *N*-acetyl galactosamine.

The above carbohydrate structure is common in mucin-type glycoproteins that are expressed widely on human normal cells and tumours (Sutherland et al, 1988; Corraway and Hull, 1989; Lesuffleur et al, 1994). Therefore, the remarkable specificity of mAb M2A, D1-26 and D2-40 for fetal gonocytes, CIS and germ cell tumours is likely based on the recognition by these mAb of a mixed epitope whose structure is determined by the polypeptide core together with the O-linked carbohydrate chain.

Two models have been proposed for the progression of testicular CIS to invasive TGCT. In the independent progression model, CIS can progress directly to SE and EC. In the linear progression model, SE is an intermediate stage between CIS and EC. In both models, EC is believed to be the precursor of IT and MT, YS and CC. Our results do not distinguish between the two models, but demonstrate a reprogramming in the expression of M2A antigen during the transition of either CIS or SE to EC, and of EC to IT, MT, YS and CC. Specifically, although there was uniform cell membrane expression of M2A antigen in CIS and SE, there was either no expression in EC, or focal membrane expression (Figure 1, Table 1). Furthermore, while this focal pattern of expression was common to all non-seminomatous TGCT that expressed M2A antigen, complete absence of expression was seen more frequently in IT, MT, YS and CC than in EC (Table 1). This is compatible with the notion that progressive loss of M2A antigen expression occurs when EC becomes transformed into the other non-seminomatous TGCT types.

M2A antigen belongs to the set of antigenic and molecular markers that are useful to follow the transformation of fetal gonocytes to CIS and the progression of CIS to invasive TGCT. At present, the elucidation of the carbohydrate epitope of M2A antigen does not allow us to make any functional correlations between the structure of the epitope and the above transitions. However, such a correlation may be possible when additional information about critical glycosylation reactions and mechanisms of receptor activation influencing these becomes available. In addition, the availability of mAb D2-40 which reacts with M2A antigen in conventionally preserved surgical specimens, opens up the way to examining archival collections of tissues for the expression of this antigen and further correlating this expression with milestones in the origin and progression of germ cell neoplasia.

### ACKNOWLEDGEMENTS

This work was supported by the National Cancer Institute of Canada with funds from the Canadian Cancer Society. JI was the recipient of a CH Best Postdoctoral Fellowship. We wish to thank Miss Lynn Wang and Mr Andrew Chong for excellent technical assistance, Dr Laurence Becker for providing specimens of pineal seminoma, and Dr Inka Brockhausen for helpful discussion.

### REFERENCES

Bailey D, Baumal R, Law J, Sheldon K, Kannampuzha P, Stratis M, Kahn H and Marks A (1986) Production of a monoclonal antibody specific for seminomas and dysgerminomas. *Proc Natl Acad Sci USA* **83**: 5291–5295

Bailey D, Baumal R and Marks A (1988) Use of anti-seminoma monoclonal antibody to confirm the diagnosis of mediastinal seminoma. *APMIS* **96**: 206–210

Bailey D, Marks A, Stratis M and Baumal R (1991) Immunohistochemical staining of germ cell tumours and intratubular malignant germ cells of the testis using antibody to placental alkaline phosphate and a monoclonal anti-seminoma antibody. *Modern Pathol* **4**: 167–171

Baumal R, Bailey D, Giwercman A, Skakkebæk N, Stratis M and Marks A (1989) A novel maturation marker for human Sertoli cells. *Int J Androl* **12**: 354–359

Buckman R, De Angelis C, Shaw P, Covens A, Osborne R, Kerr I, Reed R, Michaels H, Woo M, Reilly R, Law J, Baumal R, Groves E and Marks A (1992) Intraperitoneal therapy of malignant ascites associated with carcinoma of ovary and breast using radioiodinated monoclonal antibody 2G3. *Gyn Oncol* **44**: 102–109

Corraway KL and Hull SR (1989) O-glycosylation pathway for mucin-type glycoproteins. *BioEssays* **10**: 117–121

Giwercman A, Marks A, Bailey D, Baumal R and Skakkebæk NE (1988) A monoclonal antibody as a marker for carcinoma-in-situ germ cells of the human adult testis. *APMIS* **96**: 667–670

Giwercman A, Cantell L and Marks A (1991) Placental-like alkaline phosphatase as a marker of carcinoma-in-situ of the testis. Comparison with monoclonal antibodies M2A and 43–9F. *APMIS* **99**: 586–594

Giwercman A, Andrews PA, Jørgensen N, Müller J, Græm N and Skakkebæk NE (1993) Immunohistochemical expression of embryonal marker TRA-1–60 in carcinoma in situ and germ cell tumors of the testis. *Cancer* **72**: 1308–1314

Goss PE, Schwertfeger L, Blackstein ME, Iscoe NA, Ginsberg RJ, Simpson WJ, Jones DP and Shepherd FA (1994) Extragonadal germ cell tumors. *Cancer* **73**: 1971–1979

Jacobson GK and Norgaard-Pedersen B (1984) Placental alkaline phosphatase in testicular germ cell tumours and in carcinoma-in-situ of the testis. *Acta Pathol Microbiol Scand (A)* **92**: 323–329

Jørgensen N, Rajpert-De Meyts E, Græm N, Müller J, Giwercman A and Skakkebæk N (1995) Expression of immunohistochemical markers for testicular carcinoma in situ by normal human fetal germ cells. *Lab Invest* **72**: 223–231

King BL, Peng H-Q, Goss P, Huan S, Bronson D, Kacinski BM and Hogg D (1997) Repeat expansion detection analysis of (CAG)n tracts in tumor cell lines, testicular tumors, and testicular cancer families. *Cancer Res* **57**: 209–214

Lesuffleur T, Zweibaum A and Real FX (1994) Mucins in normal and neoplastic human gastrointestinal tissues. *Crit Rev Oncol Hematol* **17**: 153–180

Meng FJ, Zhou Y, Skakkebæk NE, Marks A and Giwercman A (1996) Detection and enrichment of carcinoma in situ cells in semen by an immunomagnetic method using monoclonal antibody M2A. *Int J Androl* **19**: 365–370

Nicholson PW and Harland SJ (1995) Inheritance and testicular cancer. *Br J Cancer* **71**: 421–425

Peters BP, Ebisu S, Goldstein IJ and Flashner M (1979) Interaction of wheat germ agglutinin with sialic acid. *Biochemistry* **18**: 5505–5511

Plummer TH, Elder JH, Alexander S, Phelan AW and Tarentino AL (1984) Demonstration of peptide:*N*-glycosidase F activity in endo-β-*N*-acetylglucosaminidase F preparations. *J Biol Chem* **259**: 10700–10704

Rajpert-De Meyts E and Skakkebæk NE (1994) Expression of the *c-kit* product in carcinoma-in-situ and invasive testicular germ cell tumours. *Int J Androl* **17**: 85–92

Rajpert-De Meyts E, Kvist M and Skakkebæk NE (1996) Heterogeneity of expression of immunohistochemical tumour markers in testicular carcinoma in situ: pathogenetic relevance. *Virchows Arch* **428**: 133–139

Schneider C, Newman RA, Sutherland DR, Asser U and Greaves MF (1982) A one-step purification of membrane proteins using a high efficiency immunomatrix. *J Biol Chem* **257**: 10766–10769

Sheldon K, Marks A and Baumal R (1986) Characterization of binding of four monoclonal antibodies to the human ovarian adenocarcinoma cell line HEY. *Biochem Cell Biol* **65**: 423–428

Skakkebæk NE and Berthelsen JG (1981) Carcinoma-in-situ of the testis and invasive growth of different types of germ cell tumours. A revised germ cell theory. *Int J Androl* **4**: 26–34

Skakkebæk NE, Berthelsen JG, Giwercman A and Müller J (1987) Carcinoma-in-situ of the testis: possible origin from gonocytes and precursor of all types of germ cell tumours except spermatocytoma. *Int J Androl* **10**: 19–28

Strohmeyer T, Peter S, Hartmann M, Munemitsu S, Ackermann R, Ullrich A and Slamon DJ (1991) Expression of the *hst*-1 and *c-kit* protooncogenes in human testicular germ cell tumours. *Cancer Res* **51**: 1811–1816

Sutherland DR, Rudd CE and Greaves MF (1984) Isolation and characterization of a human T lymphocyte-associated glycoprotein (gp 40). *J Immunol* **133**: 327–333

© Cancer Research Campaign 1999

Sutherland DR, Watt SM, Dowden G, Karhi K, Baker MA, Greaves MF and Smart JE (1988) Structural and partial amino acid sequence analysis of the human hemopoietic progenitor cell antigen CD34. *Leukemia* **2**: 793–803

Tarentino AL and Maley F (1974) Purification and properties of an endo-β-*N*-acetylglucosaminidase from *Streptomyces griseus*. *J Biol Chem* **249**: 811–816

Von der Maase H, Rørth M, Walbom-Jørgensen S, Sørenson BL, Strøyer Christopherson IS, Hald T, Krag Jacobsen G, Berthelsen JG and Skakkebæk NE (1986) Carcinoma-in-situ of contralateral testis in patients with testicular germ cell cancer: study of 27 cases in 500 patients. *Br Med J* **233**: 1398–1601

Von der Maase H, Giwercman H, Müller J and Skakkebæk NE (1987) Management of carcinoma-in-situ of the testis. *Int J Androl* **10**: 209–220

Wu AM, Sugii S and Herp A (1988) A guide for carbohydrate specificities of lectins. In *Molecular Immunology of Complex Carbohydrates*, Wu AM (ed), pp. 819–847. Plenum Press: New York.

*© Cancer Research Campaign 1999*
